# Digestive Malacoplakia in Children: Case Report

**DOI:** 10.5402/2011/597350

**Published:** 2010-12-28

**Authors:** J. Bouguila, K. Brahim, M. Mokni, K. Skandrani, A. Harbi, A. S. Essoussi, L. Boughammoura

**Affiliations:** ^1^Paediatrics Department, Hospital Farhat Hached, 4000 Ibn El Jazzar Street, Sousse, Tunisia; ^2^Laboratory of Anathomopathology, Hospital Farhat Hached, Sousse, Tunisia; ^3^Cabinet Gastroenterology, Street Léopold Senghor, Sousse, Tunisia; ^4^Paediatrics Department, Hospital Sahloul, Sousse, Tunisia

## Abstract

Malacoplakia is a form of chronic granulomatous inflammatory reaction that rarely affects the pediatric age group. The gastrointestinal system is the second most common site for the occurrence of malacoplakia. We report the case of a 9-year-old girl who was hospitalized for abdominal pain, chronic diarrhea, and rectal hemorrhage. The endoscopic examinations and histopathology confirmed the diagnosis of intestinal malacoplakia. We successfully treated her with oral levofloxacin. This disease does not have any specific clinical or biological signs, and the diagnosis is exclusively based on histology.

## 1. Introduction

Malacoplakia is an acquired granulomatous disorder first described by Michaelis and Gutmann in 1902 [1]. It's believed to be caused by an alteration of the bacterial phagocytic system [2]. Malacoplakia has been described in numerous anatomic locations, most commonly in the genitourinary tract. The gastrointestinal system, especially the colon, is a second common site for the occurrence of malacoplakia [3]. Clinically, it can mimic a neoplasm that occurs in the form of a mass [4]. The diagnosis is exclusively based on histology. The cardinal finding is the presence of numerous histiocytes with pathognomonic intracellular and extracellular Michaelis-Gutmann bodies [5].

## 2. Case Report

A 9-year-old girl presented with history of abdominal pain, prolonged diarrhea, and rectal bleeding. She presented cutaneous leishmaniasis at 3-year-old, diffuse verruca vulgaris, recurrent skin, and respiratory infections. 

She was hospitalised two years ago for pneumonia. Physical examination found hepatosplenomegaly and cervical adenopathy. The only abnormal laboratory test was an increase of *γ*-globulin count at 26,9 g/L. Inflammatory and infectious biological tests were normal. The humoral and cellular immunity was normal.

Ultrasonography and computed tomographic scan of the chest and abdomen showed numerous mesenteropelvic and inguinal gonglions and homogenous hepatosplenomegaly. The liver and the spleen had a size of 19 cm and 12 cm respectively.

Biopsy of an adenopathy showed a non specific mixed follicular and paracortical hyperplasia, with by place eosinophil's infiltration. The liver biopsy was normal.

The patient was lost to followup, and she was rehospitalized after two years. The physical examination revealed a pale, cachectic girl whose spleen was palpable 2 cm below the left costal margin and the liver had a size of 12 cm. She had a numerous superficial lymph nodes measuring 1 cm. The rectal examination ached and revealed some blood.

The laboratory workup showed a normal leukocyte count at 9700/mm^3^ with hypereosinophilia at 1700/mm^3^. The haemoglobin and the platelet counts were normal. The erythrocyte sedimentation rate was 26 mm/hour, and the *γ*-globulin was increased to 26,5 g/L. The biologic hepatic and renal functions were normal. 

We completed during this hospitalization the exploration with endoscopic examinations. The gastroscopy revealed a normal stomach, multiple polypoid lesions measuring 0.2 cm, and friable mucosa in duodenum. Multiple biopsy specimens were taken. Microscopic examination showed lymphoid hyperplasia.

**Figure 1:**
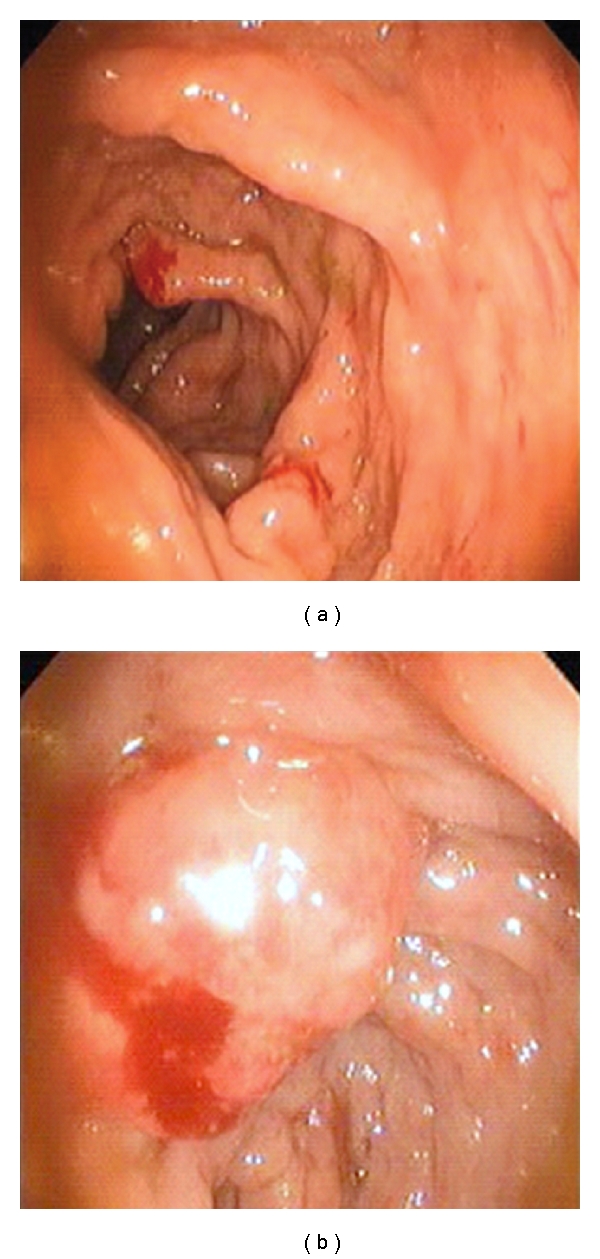
(a) Endoscopic view showing an extended colitis with edema and thickening of mucosa, (b) bleeding rectal polyp.

The colonoscopy and rectoscopy revealed an extended colitis with superficial ulcerations, friable mucosa, and bleeding rectal polyps measuring 2 cm (Figure 1(a) and 1(b)).

**Figure 2:**
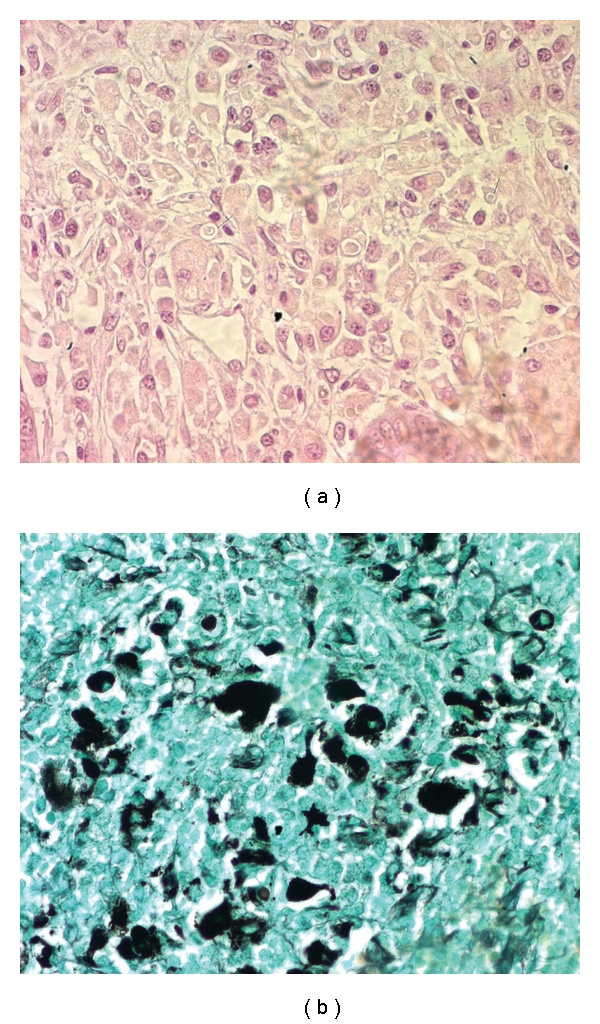
(a) Diffuse infiltration of the colonic mucosa by numerous histiocytes with cy toplasmic inclusions, (b) lesion which stain positively with periodic acid-Schiff, Von Kossa, and Prussian blue stains: Michaelis-Gutmann bodies.

The histopathological study showed a diffuse infiltration of the colonic mucosa by numerous histiocytes with cytoplasmic inclusions (Figure 2(a)), which stain positively with periodic acid-Schiff, Von Kossa, and Prussian blue stains (Figure 2(b)). Histiocytes had characteristic Michaelis-Gutmann bodies. The final diagnosis was malacoplakia with primary involvement of the gastrointestinal system.

**Figure 3:**
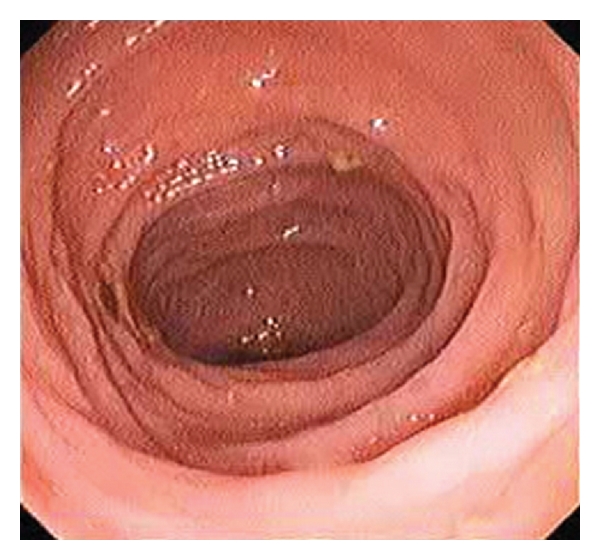
Endoscopic view after two years of treatment showing a normal intestinal mucosa.

The patient was treated with oral fluoroquinolone (Ciprofloxacin) 10 mg/Kg × 2/day. The patient's course was uneventful, and she was followed well (5 months after starting treatment). Diarrhea and rectal bleeding had stopped and hepatosplenomegaly decreased. The colonoscopy was controlled after two years of antibiotic treatment; we found that infiltration and ulceration of intestinal mucosa had decreased with a normal rectum (Figure 3). The histologic examination showed a non specific inflammation without Michaelis-Gutmann bodies.

Actually, the patient has a normal growth. The hepatosplenomegaly was present with normal biologic tests. The follow up is of a three-year period.

## 3. Comments

Malacoplakia is a rare, histologically unique, chronic inflammatory reaction of various organs such as the urinary tract and gastrointestinal system [3, 4]. The term “malacoplakia”, meaning “soft” and “plaque” in Greek, was coined by Von Hansemann in 1903 to describe yellowish tumor-like lesion in the urinary bladder [3, 6]. Since 1902, approximately 400 cases of malacoplakia have been reported in the literature [7]. 

There is a bimodal age incidence, with one peak for children below 13 years and second peak for middle-aged adults [5]. The mean age at diagnosis was around the fifth decade of age [2]. Malacoplakia is rare in children and usually involves the gastrointestinal tract, and is associated with significant additional systemic disease [8]. A female-to-male preponderance of 4 : 1 is reported when the disease affects genitourinary system [2, 7]. In the other locations, male and female patients were equally affected [7]. 

Some authors emphasize the correlation between immunosuppression and malacoplakia, because an increasing number of cases in immunodeficient individuals and in patients receiving chemotherapy, has been reported [5, 6]. Furthermore, malacoplakia has been correlated with malignant neoplasms, systemic diseases, mycotic infections, liver diseases, sarcoidosis, ulcerative colitis, cachexia, and drug addiction [5]. In our case, the patient presented in her clinical history a cutaneous leishmaniasis, diffuse verruca vulgaris with recurrent skin, and respiratory infections, but the humoral and cellular immunity were normal.

The malacoplakia may affect many organs, but frequently involves the urinary tract in 75% of cases [3, 4]. Other locations were also described including the gastrointestinal system, skin, soft tissue lung, bone, retroperitoneum, spleen, pancreas, lymph nodes, brain, and liver [4, 5, 7]. The gastrointestinal tract being the second most frequent location outside the urogenital tract; gastrointestinal and retroperitoneal sites were each identified in 12% of cases [5]. In the majority of cases, the colorectum was the commonly involved part [7]. Colonic malacoplakia was first described by Terner and Lattes in 1965 [4, 5]. The descending colon, sigmoid, and rectum are common sites and can also involve the upper tract [4]. In our case, the entire colon, the rectum, and the duodenum were involved.

Clinical manifestations of colonic malacoplakia are nonspecific; rectal bleeding, diarrhea, abdominal pain, and fever are the usual presenting symptoms [5, 9]. The endoscopy can reveal three different gross morphologic patterns reported in cases of gastrointestinal malacoplakia; unifocal mucosal, widespread multinodular or polypoid, and large mass involving adjacent organs [3]. In the present case, the endoscopy revealed superficial ulcerations, friable mucosa, and bleeding rectal and duodenal polyps.

Clinical presentation and endoscopic lesions, such unifocal nodules and polypoidal or mass lesions, can often mimics a mistaken diagnosis of ulcerative colitis and carcinoma [3, 4]. The definitive diagnosis is exclusively based on histopathologic examination; the cardinal finding is the presence of numerous histiocytes with pathognomonic intracellular and extracellular Michaelis-Gutmann bodies. Theses bodies stain positively with periodic acid-schiff, von Kossa, and Prussian blue stains [3, 5].

Even after a century since the lesion was first described, the pathogenesis of malacoplakia remains unclear [4]. The condition may result from incomplete bacterial killing and digestion by phagocytic mononuclear cells, which leads to accumulation of mononuclear cells or “Von Hansemann cells” containing partially digested bacterial fragments [10, 11]. Incomplete bacterial killing and phagolysosomes with subsequent deposits of iron and calcium constitutes inclusions known as Michaelis-Gutmann bodies [9, 12].

 The microorganisms most commonly cultured from malacoplakia are gram-negative rods *(Escherichia Coli, Klebsiella*, and *Enterobacter*) and gram-positive cocci *(Staphylococcus aureus*, *Enterococcus, and *several varieties of *Streptococcus*) [13]. This postinfectious hypothesis was based on constatation that 90% of malacoplakia involving genitor-urinary tract were associated with coliform infection, and in 72% of cases the microorganism was an *Escherichia Coli* [14].

It has been suggested that there is an abnormal macrophage response with defective lysosomal function [4]. Some studies have demonstrated low levels of cyclic guanosine monophosphate in the monocytes of these patients [5]. This decreased level may interfere with microtubular and lysosomal activity, leading to incomplete elimination of bacteria from macrophages [4]. In conclusion, the pathogenesis of malacoplakia includes two factors: bacterial infection and disorder of immunologic system. In the present case, the patient had a history of cutaneous leishmaniasis and recurrent infections, but the immunity exploration was normal.

Because malacoplakia of the gastrointestinal tract is rare, treatment is based mainly on anecdotal reports. Therapy has been both pharmacologic and surgical [3]. Medical therapy consists of treatment with antibiotics, using drugs that easily permeate the macrophage and destroy undigested bacteria such as quinolones, rifampicin, and trimethoprim-sulfamethoxasole [4, 15, 16]. Actually, Ciprofloxacin is preferred with approximately 90% of success versus 10% with trimethoprim-sulfamethoxazole [5, 6]. Cholinergic agents, such as bethanechol, were used in order to raise intracellular levels of cyclic guanosine monophosphate in macrophages. This conservator treatment is indicated when the involved organ has been not definitively undamaged.

Although the response to therapy is unpredictable, patients may respond if the treatment is continued on a long-term basis, in order to prevent the recurrence and the extension of diseases [6]. In our case, the child was treated with oral fluoroquinolone with a clinical and histological improvement after two years of treatment. In cases of progressive lesion or no response to medical treatment, the surgical resection may be the other choice of treatment [4–6].

## 4. Conclusion

Malacoplakia is rarely reported in children and usually involves the gastrointestinal tract in this age. This disease does not have any specific clinical or laboratory signs and the diagnosis is exclusively based on histology. The intestinal malacoplakia may be more common than usually suspected, especially in immunodeficient patients. It must be ruled out in every patient having chronic bloody and mucous diarrhea.
